# Perceived Discrimination and Binge Eating Disorder; Gender Difference in African Americans

**DOI:** 10.3390/jcm7050089

**Published:** 2018-04-24

**Authors:** Shervin Assari

**Affiliations:** 1 Department of Psychiatry, University of Michigan, 1415 Washington Heights, Ann Arbor, MI 48109-2029, USA; assari@umich.edu; Tel.: +1-734-647-7944; 2 Center for Research on Ethnicity, Culture and Health, School of Public Health, University of Michigan, 1415 Washington Heights, Ann Arbor, MI 48109-2029, USA

**Keywords:** perceived discrimination, Binge Eating Disorder, race, gender

## Abstract

Environmental stressors, such as perceived discrimination (PD), are linked to Binge Eating Disorder (BED). The current study investigated the association between PD and BED among African Americans, and the variation in such an association based on gender. Data of the National Survey of American Life (NSAL), 2001–2003, with a nationally-representative sample of African American adults, were used (*n* = 3516). The independent variable in the study was PD. The dependent variable was BED, measured using the Composite International Diagnostic Interview (CIDI). Socio-demographics (age, education, employment, and marital status) were covariates, and gender was the moderator variable. Survey logistic regressions with and without gender × PD interaction terms were used for data analysis. In the pooled sample, PD was associated with higher odds of BED, net of socio-demographic factors. Models also showed a significant gender × PD interaction term suggesting a stronger association between PD and BED for women, compared to men. Gender specific models showed an association between PD and BED among female, but not male, African Americans. Although a link may exist between PD and BED among African Americans, the magnitude of this association depends on gender, with a stronger association among females than males. This finding is in line with the literature that has shown gender-specific consequences of environmental stress for African Americans.

## 1. Introduction

With the current epidemics, obesity is one of the most pressing national public health challenges in the United States (US) [1,2,3,4]. As obesity is a risk factor for hypertension [5], diabetes [6], cardio-metabolic conditions [7], stroke [8], and cancer [9] it imposes enormous costs to the health care system and society. The presence of obesity, as well as time lived with obesity, both increase the risk of cardiovascular death [10]. Individuals with obesity have a higher need for physician visits, hospitalizations, diagnostic procedures, medical interventions, medications, and work loss [11,12]. The US currently spends about $190 billion dollars on obesity-related health care expenses on an annual basis [13]. Such expenditures exceed those related to smoking and problem alcohol use [14].

In addition to the economic burden to the society, obesity is also a major contributor to the existing racial disparities in health of Americans [15]. Compared to Whites, African Americans are at a 50% increased risk of obesity [16]. Obesity is more common in African American women than any other race by gender group [16]. With four of five being overweight or obese, the risk of obesity for African American women is 80% [16].

Binge Eating Disorder (BED) are associated with obesity [17,18]. As newly-regarded as a psychiatric disorder by the American Psychiatric Association Diagnostic and Statistical Manual of Mental Disorders-5 (DSM-5). BED’s key diagnostic feature is recurrent and persistent episodes of binge eating, accompanied by a loss of control. BED is commonly associated with binge eating, emotional eating, and over-eating [19], which all change the energy balance by increasing the energy input [20], and may result in accumulation of body fat [21]. According to some reports, BED is more common in women than men [22], and BED are possibly more salient determinants of obesity for women than men [23]. BED is also correlated with obesity in low socioeconomic status African American women [24].

Environmental stressors such as perceived discrimination (PD) are causally linked to binge eating [24,25,26,27,28,29,30] and BED [31,32,33,34]. Altered stress response is documented in individuals with BED that causes difficulties in coping with stressors among individuals with BED [35]. In line with the other observed gender differences in BED and associated obesity [36,37,38,39], environmental stressors, such as PD, may differently contribute to BED for African American males and females [34,40,41].

It is still unknown, however, whether the association between PD and BED among African Americans depend on gender. Environmental stressors are more salient determinants of obesity for African American females than males [22,42]. This is in contrast to the stronger effects of environmental stressors such as PD on psychological distress [43], anxiety [44], depression [44,45], and substance use [46,47,48], for males than females.

To respond to the knowledge gap regarding the link between PD and BED among African Americans, the current study was performed with two aims: (1) to investigate the association between PD and BED; and (2) to explore gender differences in this association. Regarding the first aim, high PD was expected to be associated with higher odds of BED. In line with the literature on gender variation in correlates of BED [40,49,50], gender differences were hypothesized in the association between PD and BED, with stronger effect for female than male African Americans. To generate nationally-representative results, this study borrowed data from the National Survey of American Life (NSAL), a national survey with a representative sample of African Americans in the United States [51]. The results are hoped to provide insight to prevent obesity in African Americans, particularly African America women [52,53].

## 2. Methods

### 2.1. Design and Setting

Using a cross-sectional design, the current study borrowed data from the NSAL. NSAL was a nationally-representative survey conducted by the University of Michigan between February 2001, and June 2003 [51,54]. As the primary aim of the NSAL was to assess epidemiology of psychiatric disorders, all participants in the NSAL were interviewed using the Composite International Diagnostic Interview (CIDI) diagnostic interview [55,56]. As a result, NSAL generated epidemiological data regarding major non-psychotic psychiatric disorders.

### 2.2. Ethics

The NSAL study protocol received approval by the University of Michigan Institute Review Board. All participants provided informed written consent for their participation.

### 2.3. Participants

The NSAL included Non-Hispanic Whites and Non-Hispanic Blacks. The original sample included 5810 individuals including 3516 African Americans, 1415 Caribbean Blacks, and 879 Non-Hispanic Whites. The current analysis only included 3516 African Americans, given the focus was exclusively on African Americans.

The NSAL study used a national household probability sampling strategy. Individuals were eligible if they were 18 years and older [25,26], were not institutionalized, and were able to speak English. African Americans in the NSAL were sampled from large cities, other urban areas, or rural areas.

### 2.4. Interview

Data were collected through either face-to-face (86%) or telephone (14%) interviews. Face-to-face interviews were computer-assisted personal interviews (CAPI). Each interview lasted 140 min on average. Interviews were performed in English. The final response rate was 70.7% for the African American sample.

### 2.5. Measures

#### 2.5.1. Gender

Gender was the moderator variable, which was operationalized as a dichotomous variable (male 0 (reference category) vs. female 1).

#### 2.5.2. Sociodemographic Factors

Socio-demographics in this study included age, employment status, education attainment, household income, marital status, and country region. Employment status, marital status, and country region were conceptualized as categorical variables. Country region was a four-level categorical variable (Northeast (reference category), Midwest, South, and West). Education attainment and household income were operationalized as continuous measures. Education attainment was the number of education years completed, and household income was in dollars.

#### 2.5.3. Obesity

Obesity was defined based on the body mass index (BMI) which was calculated based on self-reported weight and height. Although BMI based on self-reported data may lead to under-estimation of obesity [57], BMI based on self-reported data is highly correlated with BMI based on direct measures [55,58]. Obesity was defined as BMI 30 or more [59,60].

#### 2.5.4. Perceived Discrimination (PD)

Perceived everyday discrimination was measured using the 10-item Everyday Discrimination Scale (EDS), developed by Williams, Jackson, and Anderson in 1997 [61]. The measure evaluates the subtle unfair treatment on day-to-day basis that individuals encounter on a daily basis. This measure has 10 items that are on a 6-point Likert scale (1 = never to 6 = almost every day). Some of the example items include: (1) “You are treated with less courtesy than other people”; (2) “You are followed in stores”; and (3) “You are called names or insulted”. An average score was calculated, with higher scores representing a higher frequency of everyday discrimination. α = 0.89 for African Americans.

#### 2.5.5. Binge Eating Disorders

Using the CIDI diagnostic interview [55,56], all participants were first asked the following screener item “Ever have a strong fear/concern about being too overweight”. Individuals who endorsed a positive history of “strong fear/concern about being too overweight” were asked all the questions in the CIDI eating disorder module. The CIDI binge-eating module asks the participants several items that are based on the DSM-IV-TR definition of BED (1) sense of loss of control over eating; and (2) eating a large amount of food. Positive responses to both items were required for a possible BED diagnosis. Participants were also asked whether the binge episodes were associated with any of the following issues: (1) eating until uncomfortably full; (2) eating more rapidly; (3) eating when not physically hungry; (4) eating alone because being embarrassed by the amount of food eaten; and (5) feeling guilty/depressed/disgusted after overeating. At least three positive endorsements from the above items were required for a diagnosis of BED. According to the DSM-IV-TR, in BED, binge eating should cause marked distress, cannot occur during the course of another eating disorder, and cannot be associated with weight-related compensatory behaviors. Algorithms were created to define BED consistent with the DSM-IV-TR. Inter-rater κ for the BED is about 0.80 [62,63,64].

### 2.6. Statistical Analysis

To analyze the data, Stata 13.0 (Stata Corp., College Station, TX, USA) was used. As mentioned before, NSA has used a multistage sampling design that involved clustering and stratification. To accommodate the NSAL complex sampling design, statistical techniques were applied to account for the sampling weights based on strata, clusters, and non-response. Sub-population commands were used for all the data analysis. Survey proportions and means with their 95% confidence intervals (CI) were used to describe the sample. Logistic regression was used for multivariable analysis. PD was the predictor, BED was the outcome, sociodemographic factors were the covariates, and gender was the moderator. Four regression models were estimated. *Model 1* and *Model 2* were performed in the pooled sample, while *Model 3* and *Model 4* were in males and females, respectively. *Model 1* did not include any interaction term. *Model 2* also included gender by PD interaction term. Odds ratio (OR), 95% CI, and *p* values were reported. *p* values less than 0.05 were considered statistically significant. *p* values less than 0.1 were considered marginally significant.

## 3. Results

This study included 3516 African Americans who identified as male (*n* = 1271) or female (*n* = 2299). Compared to men, women reported lower PD. Women also had a higher prevalence of obesity. Men and women did not differ in prevalence of BED. Table 1 presents the descriptive statistics for the total sample, as well as males and females.

### 3.1. Model 1

Based on *Model 1*, in the pooled sample, PD was associated with higher odds of BED (OR = 1.36; 95% CI = 1.00–1.86, *p* < 0.05), net of demographics, socioeconomic status, and region. In the pooled sample, high PD was associated with higher odds of BED (Table 2).

### 3.2. Model 2

*Model 2*, which was performed in the pooled sample, showed a main effect of PD on BED (OR = 1.72; 95% CI = 1.39–2.12, *p* < 0.05). This model also showed a significant gender × PD interaction term (OR = 1.48; 95% CI = 1.03–2.12, *p* < 0.05) suggesting a stronger positive association between PD and BED for females, compared to males (Table 2).

### 3.3. Model 3

*Model 3* showed a marginally significant main effect of PD on BED in males (OR = 1.36; 95% CI = 0.99–1.87, *p* < 0.1), suggesting that, although not significant, higher PD had a tendency toward an association with higher odds of BED in females (Table 3).

### 3.4. Model 4

*Model 4* showed a main effect of PD on BED in females (OR = 2.04; 95% CI = 1.63–2.55, *p* < 0.05). In females, higher PD was associated with higher odds of BED (Table 3).

## 4. Discussion

The current study had two aims: (1) to investigate the association between PD and BED; and (2) to explore gender differences in this association. While PD was associated with higher odds of BED, a stronger association was found for female, compared to male, African Americans.

The first finding was in line with the past research documenting the role of environmental stressors such as interpersonal problems and stressful life events as risk factors for binge eating [24,25,26,27,28,29,30] and BED [32,33,34]. Research has also documented an altered stress response in individuals with BED, which is an indicator of chronic stress in BED [35]. The current study extends this field in two ways: First, by establishing this association for African Americans, a demographic group, which is traditionally under-represented in academic research, and second, by showing that the same association hold for PD as the type of stressor.

The second finding is also in line with some previous research on gender differences in correlates of BED and associated obesity [36,37,38,39]. Previous studies have shown that environmental stressors may be differently salient for males and females, with a more significant role for females than males [34,40,41]. In a cross-sectional study on a community sample, gender differences were found in several paths that were involved in stress and emotional eating. The association between perceived stress and emotional eating, the association between perceived stress and binge eating, and the association between binge eating and increased the homeostasis model assessment-estimated insulin resistance (HOMA-IR) were all stronger for females than males [41]. In another study, among individuals with BED, women more frequently engaged in eating in response to negative emotions (anxiety, anger, and depression) than men [40]. In another study, stress was associated with post-stress hunger and post-stress anxiety and negative affect, which, in turn, were associated with calories used for women with BED [34]. Another study of adolescents showed effects of perceived stress, worries and tension/anxiety to emotional eating for females, but not males [50]. All these suggest that stress and associated affect may be more salient components of eating for women than men with BED. These also suggest that among women, stress reduction may be an important target to decrease obesity related to binge and emotional eating and BED [41].

The observed gender difference extends the previously reported gender differences in the associations between distress, BED, obesity, body dissatisfaction, and weight loss behaviors. In a number of studies, obesity is shown to cause smaller distress for Black females than other demographic groups, particularly Whites [65,66,67,68,69,70]. At the same time, actual obesity has smaller effects on perceived obesity for Blacks, particularly Black males [71,72]. In a single study, obesity was negatively associated with risk of depression for African American males [59]. Blacks are less likely to be concerned about being overweight [73,74,75,76]. Blacks with obesity maintain more positive body image than other groups, maybe because they are more tolerant toward larger body sizes [77,78,79,80,81]. Thus, similar obesity may not be associated with similar distress and body dissatisfaction for Blacks than Whites [82,83]. Societal, structural, cultural, and behavioral factors may have role in explaining some of these racial differences [84,85,86]. The association between actual obesity and perceived obesity and weight control behaviors are also weaker for African Americans than Whites [71]. The findings of this study and also the above mentioned literature, collectively, suggest that the processes involved in shaping the risk of obesity are very different for Black males and Black females. These race and gender differences have major implications for additional risks of obesity in gender and ethnic groups of Blacks, including African American women.

### 4.1. Implications

The results have some implications for prevention of obesity in Blacks, and a reduction of the racial gap in obesity. More than 60% of the US general population is overweight or obese [21,87,88]. This is about 80% for African American women [89]. Obesity is responsible for about 300,000 deaths annually, only second to cigarette smoking [90]. With the current trend for obesity [21,91], mortality and morbidity of obesity is expected to increase even more in the near future [92]. While individual behaviors and unhealthy lifestyles are primarily blamed [16,87,88,93,94,95,96,97], discrimination and racism also have a major role. There is a need to consider a reduction of discrimination as a main strategic goal to prevent obesity in African American women [98], at least due to BED. Prevention of obesity in minorities require multi-level strategies that include policies and programs, as well as structural and individual factors [94,95,96,97].

The findings reported here may have some implications for reducing racial gap in mortality due to obesity and associated metabolic conditions. Obesity increases risk of premature mortality from cardiovascular disease, and several other causes [99]. Even small weight loss will reduce the risk of medical consequences that are seen in individuals with obesity [100]. As a result, it is very important to address discrimination for prevention and treatment of BED-related obesity in African American females.

The results of the current study also have some clinical implications for the clinical practice for BED. Discrimination should be addressed as an important psychosocial construct for African American females with BED. Clinicians and practitioners may consider enhancing coping with discrimination among African American females with BED.

### 4.2. Limitations

This study has at least three limitations. First, racial variation in validity of CIDI for measurement of BED is not known. Second, self-reported data were used to measure obesity. Third, the study did not include some other covariates as well as related constructs, such as body image dissatisfaction, into this study [101,102]. The large sample size and the nationally-representative sample were the two main strengths of the study.

## 5. Conclusions

Findings of the current study suggested that the association between PD and BED among African Americans depend on gender. PD has a stronger effect on BED among women than men. This finding is in line on the gendered paths in the consequences of environmental stress on health outcomes of African Americans.

## Figures and Tables

**Table 1:** Descriptive statistics in the pooled sample and by gender.

	All			Males			Females		
	Mean	SE	95% CI	Mean	SE	95% CI	Mean	SE	95% CI
Age	42.04	0.51	40.99–43.08	41.76	0.65	40.44–43.09	42.25	0.56	41.11–43.38
Household Income (USD 10000) *	3.63	0.13	3.35–3.90	4.18	0.19	3.79–4.57	3.19	0.11	2.97–3.42
Perceived Discrimination *	1.25	0.03	1.19–1.31	1.38	0.05	1.28–1.47	1.16	0.03	1.11–1.21
	%	SE	95% CI	%	SE	95% CI	%	SE	95% CI
Gender									
Male	0.45	0.01	0.43–0.47						
Female	0.55	0.01	0.53–0.57						
Education									
0–11 years	0.24	0.01	0.22–0.27	0.23	0.02	0.20–0.26	0.26	0.02	0.22–0.29
12 years	0.38	0.01	0.36–0.40	0.40	0.02	0.36–0.43	0.36	0.01	0.34–0.39
13–15 years	0.24	0.01	0.22–0.26	0.23	0.02	0.20–0.27	0.24	0.01	0.22–0.27
16+	0.14	0.01	0.12–0.17	0.15	0.02	0.12–0.18	0.14	0.01	0.12–0.16
Employment *									
Employed	0.90	0.01	0.88–0.91	0.91	0.01	0.89–0.93	0.89	0.01	0.87–0.90
Unemployed	0.10	0.01	0.09–0.12	0.09	0.01	0.07–0.11	0.11	0.01	0.10–0.13
Marital Status *									
Unmarried	0.58	0.01	0.56–0.60	0.50	0.02	0.47–0.54	0.64	0.01	0.62–0.67
Married	0.42	0.01	0.40–0.44	0.50	0.02	0.46–0.53	0.36	0.01	0.33–0.38
Region									
Northeast	0.16	0.01	0.14–0.18	0.16	0.02	0.13–0.20	0.16	0.01	0.14–0.19
Midwest	0.17	0.01	0.15–0.21	0.16	0.02	0.13–0.20	0.19	0.02	0.16–0.22
South	0.57	0.02	0.52–0.61	0.57	0.03	0.52–0.63	0.56	0.02	0.52–0.61
West	0.10	0.01	0.08–0.12	0.11	0.01	0.09–0.13	0.09	0.01	0.07–0.11
Obesity *									
No	0.65	0.01	0.63–0.66	0.71	0.01	0.68–0.73	0.60	0.01	0.57–0.62
Yes	0.35	0.01	0.34–0.37	0.29	0.01	0.27–0.32	0.40	0.01	0.38–0.43
BED									
No	0.95	0.00	0.94–0.96	0.96	0.01	0.94–0.97	0.94	0.00	0.93–0.95
Yes	0.05	0.00	0.04–0.06	0.04	0.01	0.03–0.06	0.06	0.00	0.05–0.07

* *p* < 0.05.

**Table 2:** Association between perceived discrimination and BED among African Americans.

	*Model 1* All		*Model 2* All	
	OR	95% CI	OR	95% CI
Gender				
Male	1		1	
Female	1.38	0.87–2.18	0.70	0.37–1.32
Age	1	0.99–1.02	1	0.99–1.02
Education				
0–11 Years	1.57	0.64–3.87	1.57	0.64–3.85
12 Years	1.98	0.86–4.55	2.01 #	0.88–4.60
13–15 Years	1.19	0.45–3.17	1.21	0.46–3.19
16+	1		1	
Employment Status				
Employed				
Unemployed	1.53	0.91–2.55	1.55 #	0.92–2.63
Marital Status				
Unmarried	1		1	
Married	0.8	0.51–1.24	0.78	0.50–1.22
Region				
Northeast	0.44 *	0.21–0.89	0.43 *	0.20–0.90
Midwest	0.96	0.52–1.78	0.95	0.50–1.82
South	0.44 *	0.23–0.84	0.44 *	0.23–0.84
West	1		1	
Household Income (USD 10,000)	0.95	0.85–1.07	0.95	0.85–1.07
Obesity				
Yes	2.15 ***	1.45–3.19	2.17 ***	1.45–3.23
No	1		1	
Perceived Discrimination	1.72 ***	1.39–2.12	1.36 *	1.00–1.86
Perceived Discrimination * Female			1.48 *	1.03–2.12
Intercept	0.02 ***	0.00–0.07	0.03 ***	0.01–0.11

Logistic regression models. Outcome: Binge Eating Disorders (BED). Source: National Survey of American Life (NSAL), 2001–2003. # *p* < 0.1, * *p* < 0.05, *** *p* < 0.001.

**Table 3:** Association between perceived discrimination and BED among male and female African Americans.

	*Model 3* Males		*Model 4* Females	
	OR	95% CI	OR	95% CI
Age	1	0.97–1.02	1.01	0.99–1.02
Education				
0–11 Years	0.86	0.23–3.25	3.50 *	1.01–12.13
12 Years	1.13	0.36–3.55	4.46 *	1.22–16.27
13–15 Years	0.26	0.05–1.43	4.01 *	1.03–15.61
16+	1		1	
Employment Status				
Employed				
Unemployed	1.8	0.68–4.76	1.38	0.76–2.52
Marital Status				
Unmarried	1		1	
Married	0.8	0.45–1.40	0.80	0.43–1.51
Region				
Northeast	0.38	0.08–1.72	0.48 #	0.22–1.02
Midwest	1.44	0.39–5.31	0.80	0.37–1.72
South	0.44	0.14–1.43	0.47 *	0.24–0.90
West	1		1	
Household Income (USD 10,000)	0.97	0.84–1.13	0.93	0.78–1.10
Obesity				
Yes	1.92#	0.96–3.84	2.36 **	1.40–3.99
No	1		1	
Perceived Discrimination	1.36#	0.99–1.87	2.04 ***	1.63–2.55
Intercept	0.05 **	0.01–0.43	0.01 ***	0.00–0.05

Logistic regression models. Outcome: Binge Eating Disorders (BED). Source: National Survey of American Life (NSAL), 2001–2003. # *p* < 0.1, * *p* < 0.05, ** *p* < 0.01, *** *p* < 0.001.
